# Periorbital Structural and Functional Modulation Following Liquid-Type Polycaprolactone Treatment: A Pilot Study

**DOI:** 10.3390/jcm15114303

**Published:** 2026-06-02

**Authors:** Sung Jay Choe, Ju Hee Han, Irene Darmawan, Dong In Keum

**Affiliations:** 1 Galleria Dermatology Skin Clinic, Seoul 06526, Republic of Korea; 2 Department of Dermatology, Seoul St. Mary’s Hospital, College of Medicine, The Catholic University of Korea, Seoul 06591, Republic of Korea; alwaysmine8@gmail.com; 3 Galleria Rin Dermatology Clinic, Jakarta 12120, Indonesia

**Keywords:** periorbital rejuvenation, liquid-type polycaprolactone, collagen biostimulator, infraorbital contour, eyelid opening, functional modulation, tissue redistribution

## Abstract

**Background**: Periorbital aging involves complex structural and functional changes, including decreased eyelid opening and alterations in infraorbital contour. Conventional collagen biostimulators mainly focus on localized volume augmentation and dermal remodeling, with limited considerations for broader tissue interactions within the periorbital region. **Objective**: To evaluate structural and functional changes following treatment with a liquid-type polycaprolactone (PCL)-based collagen biostimulator. **Methods**: Three patients were analyzed. Clinical photographs were obtained at baseline, 1 month, and 3 months. Measurements were normalized to iris diameter. Eyelid-related and infraorbital contour parameters, along with canthal tilt angle (CTA), were assessed under neutral gaze and maximal effort. The treatment was performed via upper (forehead) and lower (infraorbital) approaches without direct injection into the upper eyelid. **Results**: Margin reflex distance 1 (MRD1) showed an overall increasing trend in both neutral gaze and maximal effort conditions, while ΔMRD1 remained relatively unchanged. Medial canthus-to-brow vertical distance (CBVD-M) showed a tendency to increase, whereas brow-to-lid distance (BLD) and lateral CBVD (CBVD-L) did not demonstrate consistent patterns. CTA increased across cases. Upper concavity height (UC), peak protrusion height (PP) and lower concavity height (LC) decreased concurrently. **Conclusions**: Liquid-type PCL-based treatment was associated with functional and structural changes in the periorbital region. Improved eyelid opening without direct upper eyelid injection may suggest indirect regional effects through contiguous anatomical planes. Changes in lower eyelid contour parameters support broader structural and functional changes.

## 1. Introduction

The periorbital region is among the most susceptible areas to the visible effects of facial aging, characterized by a constellation of structural changes including palpebral fissure height, altered eyebrow position, and lower eyelid fat prolapse [1,2,3].

Conventional approaches to periorbital rejuvenation have largely focused on localized volume augmentation or enhancement of dermal thickness using hyaluronic acid fillers or various collagen biostimulators [4,5,6,7]. While effective for addressing isolated structural deficits, such strategies may be insufficient to fully address the regional structural and functional changes affecting the brow–eyelid complex and the infraorbital region as a whole.

Recent anatomical and clinical studies have emphasized that periorbital aging involves coordinated changes across multiple tissue layers and support structures [8]. The forehead and periorbital region contain loose areolar connective tissue layers that may exhibit anatomical continuity, functioning as glide planes that facilitate coordinated movement between adjacent soft tissues [9,10].

Liquid-type polycaprolactone (PCL)-based collagen stimulators are characterized by their low viscosity and high diffusibility, enabling distribution along tissue planes rather than confinement to a discrete injection site [10,11]. These physical properties distinguish them from conventional volumizing agents and raise the possibility that such materials may influence regional tissue behavior beyond simple focal volume replacement by improving sliding dynamics.

Building upon this rationale, the present study investigated the structural and functional changes occurring in the periorbital region following the administration of a liquid-type PCL agent. Of particular interest was whether observable changes could be identified in the upper eyelid despite the absence of direct upper eyelid injection, which may indicate indirect regional effects through contiguous anatomical planes.

## 2. Materials and Methods

### 2.1. Study Design and Participants

This retrospective study was conducted using the data of the patients who underwent periorbital treatment with a liquid-type polycaprolactone (PCL)-based collagen stimulator (Gouri^®^, Dexlevo Inc., Seoul, Republic of Korea). Three female participants (age range: 58–65 years) with standardized serial photographs suitable for quantitative analysis were included. Exclusion criteria comprised a history of eyelid surgery, any condition affecting eyelid position or periorbital anatomy, and prior treatment with botulinum toxin or collagen-stimulating agents in the periorbital region. All patients provided written informed consent for the use of their clinical data and photographs for research and publication.

### 2.2. Treatment Protocol

All patients received injections using a mixture consisting of 1 mL of liquid-type PCL, 3 mL of normal saline, and 1 mL of 2% lidocaine with epinephrine (1:100,000), with a total volume of 5 mL administered per treatment session. Two injection approaches were employed (Supplementary Figure S1). The superior approach involved entry from the forehead above the eyebrow, with distribution directed toward the brow along the subgaleal loose areolar plane (1.5 mL). The inferior approach was performed below the lower eyelid at the tear trough region, progressing in both lateral-to-medial (0.5 mL) and inferior-to-superior (0.5 mL) directions. No direct injection was performed into the upper eyelid.

Supplementary Figure S1 was generated using an AI-assisted image generation tool (ChatGPT, OpenAI, San Francisco, CA, USA) for a schematic illustration of cannula entry points and advancement directions. The generated image was subsequently reviewed and manually edited by the authors to ensure anatomical accuracy.

### 2.3. Image Acquisition and Standardization

Clinical photographs used for the study were obtained at baseline (pretreatment), one month, and three months following the procedure. Consistent lighting conditions, camera distance, and head positioning were maintained across all time points. Patients were photographed under both neutral gaze and maximal eyelid opening conditions. To enhance measurement accuracy, all parameters were normalized to the horizontal iris diameter, which was assigned a reference value of 1.0 in each image. Image analysis was performed using ImageJ software version 1.54g (National Institutes of Health, Bethesda, MD, USA).

### 2.4. Outcome Measures

The following parameters were evaluated to assess periorbital changes:

(1) Upper Eyelid Parameters

- Margin reflex distance 1 (MRD1)
- Margin reflex distance 2 (MRD2)
- Brow-to-lid distance (BLD)
- Canthus-to-brow vertical distance (CBVD): medial (CBVD-M) and lateral (CBVD-L)

(2) Lower Eyelid Contour Parameters

- Upper concavity height (UC)
- Peak protrusion height (PP)
- Lower concavity height (LC)

(3) Angular Parameter

- Canthal tilt angle (CTA)

Primary interpretation focused on parameters derived from relatively stable landmarks, including the corneal light reflex and the medial and lateral canthi; eyebrow-based parameters were interpreted as secondary outcome variables. All distance parameters were analyzed as iris-diameter-normalized values. Definitions and measurement methodology for each parameter are described in detail in the Supplementary Materials (Supplementary Figure S2).

### 2.5. Dynamic Analysis

To evaluate functional changes, all parameters were measured under both neutral gaze and maximal eyelid opening conditions. Delta values (Δ) were calculated as the difference between measurements obtained under maximal and neutral conditions.

### 2.6. Statistical Analysis

Given the pilot nature and limited sample size, statistical analysis was primarily descriptive in nature. Changes in each parameter were compared across the baseline, one-month, and three-month time points, and temporal trends were evaluated based on normalized values. Measurement reliability analysis is described in the Supplementary Methods.

## 3. Results

**Figure 1:**
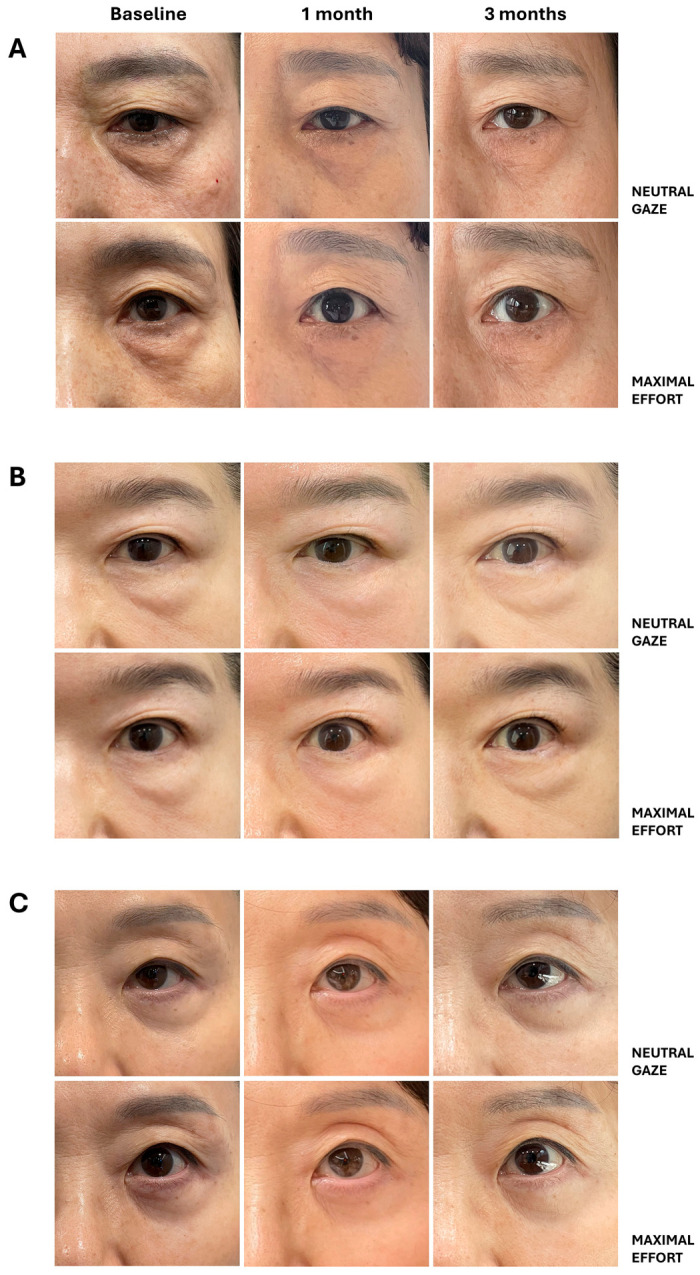
Representative clinical photographs of the periorbital region. Standardized images of the left eye in three cases at baseline, 1 month, and 3 months after treatment. Neutral gaze (upper row) and maximal effort (lower row) images are shown for each case. (**A**) Case 1; (**B**) Case 2; (**C**) Case 3.

Clinical photographs obtained at baseline, one month, and three months are presented in Figure 1 to provide a visual overview of the changes observed in each case.

### 3.1. Upper Eyelid Parameters

**Figure 2:**
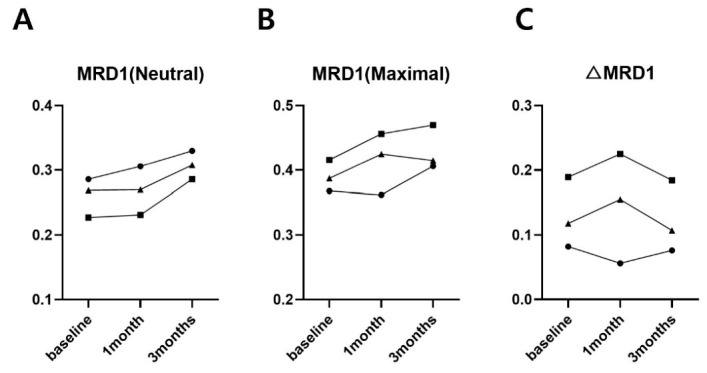
Changes in margin reflex distance 1 (MRD1). (**A**) MRD1 in neutral state, (**B**) MRD1 in maximal state, and (**C**) the difference between maximal and neutral states (△MRD1), measured at baseline, 1 month, and 3 months. All values were normalized to iris diameter. Individual cases are represented using different markers: squares for Case 1, circles for Case 2, and triangles for Case 3.

Margin reflex distance 1 (MRD1) under neutral gaze increased from baseline in all cases. Increases were observed at the one-month follow-up and were maintained or continued to increase at three months (Figure 2A). The mean percentage change (±standard deviation) was +17.6 ± 6.4%. MRD1 under maximal eyelid opening also demonstrated a general increase, relative to baseline at three months (Figure 2B); however, the magnitude of change (+9.0 ± 3.7%) was comparatively smaller than that observed under neutral gaze. In contrast, ΔMRD1—reflecting the difference between maximal and neutral states—showed no substantial change and was largely maintained over the follow-up period (Figure 2C). In addition, the relative ordering of ΔMRD1 among the three cases remained consistent across visits, indicating that inter-individual differences in dynamic eyelid excursion were preserved over time despite treatment-related changes in absolute MRD1 values.

**Figure 3:**
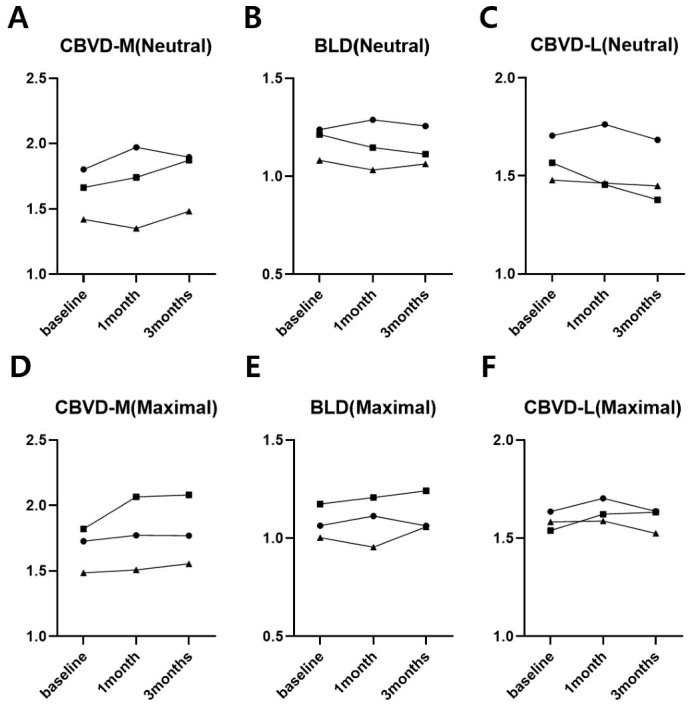
Changes in brow–eyelid relationship parameters. (**A**–**C**) Medial canthus-to-brow vertical distance (CBVD-M), brow-to-lid distance (BLD), and lateral canthus-to-brow vertical distance (CBVD-L) in neutral state, and (**D**–**F**) the same parameters in maximal state, measured at baseline, 1 month, and 3 months. All values were normalized to iris diameter. Individual cases are represented using different markers: squares for Case 1, circles for Case 2, and triangles for Case 3.

Medial canthus-to-brow vertical distance (CBVD-M) increased under both neutral and maximal conditions (Figure 3A,D). In contrast, brow-to-lid distance (BLD) and lateral canthus-to-brow vertical distance (CBVD-L) demonstrated inconsistent directional changes across cases, with both increases and decreases observed relative to baseline (Figure 3B,C,E,F).

### 3.2. Lower Eyelid Parameters

**Figure 4:**
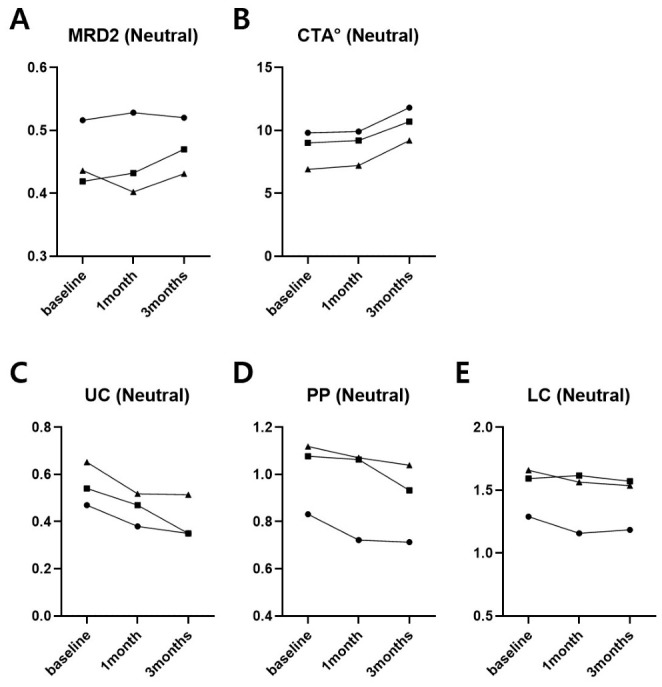
Changes in lower eyelid parameters in neutral state. (**A**) Margin reflex distance 2 (MRD2), (**B**) canthal tilt angle (CTA), (**C**) upper concavity height (UC), (**D**) peak protrusion height (PP), and (**E**) lower concavity height (LC), measured at baseline, 1 month, and 3 months. All distance values were normalized to iris diameter. Individual cases are represented using different markers: squares for Case 1, circles for Case 2, and triangles for Case 3.

Margin reflex distance 2 (MRD2) did not demonstrate a consistent pattern of change under either neutral or maximal conditions (Figure 4A).

Canthal tilt angle (CTA) exhibited a progressive increase from baseline at both the one-month and three-month time points under neutral and maximal conditions (Figure 4B). The mean change from baseline was +2.0 ± 0.5° under neutral gaze and +1.3 ± 0.5° under maximal eyelid opening.

A general trend toward reduction was observed across all lower eyelid contour parameters—upper concavity height from lower lid margin (UC), peak protrusion height from lower lid margin (PP), and lower concavity height from lower lid margin (LC)—(Figure 4C–E). UC demonstrated the most consistent reduction across all cases, with a mean decrease of −25.3 ± 10.2% from baseline. PP also decreased in all cases, with a mean reduction of −17.2 ± 7.4%. LC showed a tendency toward a decrease as well, though the magnitude was comparatively limited (−5.6 ± 3.2%). Similar reductions were observed under maximal eyelid opening conditions (Supplementary Figure S3). These reduction patterns were observed across cases under both neutral and maximal conditions and were most pronounced at the three-month time point. Detailed absolute and percentage changes from baseline to 3 months for key parameters are summarized in the table (Supplementary Table S1).

### 3.3. Reliability Analysis

Intra- and inter-rater reliability analyses demonstrated high reproducibility across all measurement parameters. Detailed reliability analysis is provided in Supplementary Table S2.

## 4. Discussion

The present pilot study quantitatively analyzed structural and functional changes in the upper and lower eyelids following treatment with a liquid-type PCL-based collagen stimulator. Rather than focusing solely on volumetric augmentation or dermal remodeling, the study aimed to explore whether changes extended beyond the directly treated areas.

Upper eyelid opening is regulated not only by levator palpebrae superioris function, but also by the elasticity of overlying skin and soft tissues, the activity of the frontalis muscle, and the sliding dynamics within the subgaleal loose areolar plane [12]. Margin reflex distance 1 (MRD1) demonstrated a general trend toward an increase under both neutral and maximal conditions (Figure 2A,B). Notably, ΔMRD1 showed no meaningful change (Figure 2C), which may indicate that the observed improvement was more evident in baseline eyelid position than in maximal opening capacity. In addition, increases in MRD1 were accompanied by clinically observable improvement in upper eyelid opening relative to the skin surface, along with a tendency toward reduction in dermatochalasis-related hooding. These findings may reflect redistribution or altered soft-tissue support rather than a direct improvement in levator function.

The liquid-type PCL formulation used in this study is characterized by relatively low viscosity and high diffusibility [10]. These properties may allow the material to distribute along low-resistance tissue planes. In this study, particular attention was given to the subgaleal loose areolar plane as a potential route of tissue-plane spread. Anatomically, this plane may extend through the sub-brow region and may communicate with the preseptal cellulofatty layer of the upper eyelid and the prezygomatic space of the lower eyelid through loose connective tissue interfaces. These layers are relatively low-resistance tissue planes within the facial region and have been described as pathways along which fluid collections such as hematoma or edema originating in the forehead, can extend toward the periorbital area under the influence of gravity [13,14].

Considering these anatomical and material properties, it is plausible that the injected material may influence interactions between tissue layers. Redistribution of the material within these planes may alter local tissue characteristics in a manner that favourably affects brow–eyelid movement during eyelid opening. This interpretation is consistent with the observed increase in baseline MRD1 without a corresponding change in ΔMRD1. In this context, the treatment may have influenced the resting configuration of the brow–eyelid complex rather than its maximal dynamic range. However, these interpretations remain hypothetical and cannot be directly confirmed by the present study. The observed improvement in MRD1 in the absence of direct upper eyelid injection may indicate effects beyond simple localized volumization and warrants further investigation. Among the brow–eyelid relationship parameters, medial canthus-to-brow vertical distance (CBVD-M) demonstrated a relatively consistent upward trend (Figure 3A,D), whereas brow-to-lid distance (BLD) and lateral canthus-to-brow vertical distance (CBVD-L) showed no uniform directional change across cases (Figure 3B,C,E,F). Although an increase in MRD1 is generally expected to be associated with a decrease in BLD, and eyebrow elevation with an increase in CBVD-L, the interaction within the brow–eyelid complex cannot be fully explained by a simple mechanical relationship. In clinical settings, the coupling between these parameters is known to be variable and not consistently observed [15]. The concurrent increase in canthal tilt angle (CTA) across all cases (Figure 4B) supports this interpretation. During aging, the medial canthal position tends to remain relatively stable while the lateral canthal complex undergoes preferential inferior displacement, which contributes to a reduction in canthal tilt [16]. The observed post-treatment increase in CTA may reflect partial attenuation of lateral canthal descent. Additionally, changes in canthal axis orientation can independently influence vertical distance-based parameters such as BLD and CBVD-L, which may explain discrepancies between visual impressions and quantitative measurements.

In the lower eyelid analysis, a general reduction was observed across upper concavity height from lower lid margin (UC), peak protrusion height from lower lid margin (PP), and lower concavity height from lower lid margin (LC) (Figure 4C–E). UC demonstrated the most pronounced and consistent decrease, while LC showed a comparatively limited magnitude of change. The simultaneous reduction in UC, PP, and LC, in conjunction with an increase in CTA, is more consistent with global infraorbital contour flattening than with selective correction of a specific anatomical subunit. In the natural aging process, infraorbital fat tends to protrude anteriorly and inferiorly due to gravity and ligamentous laxity, while the lateral canthus also descends [1,2,16]. The changes observed in this study may reflect a relative redistribution of infraorbital soft tissues in a superior and lateral direction. The limited degree of reduction in LC, compared with UC and PP, may be explained by the structural stability of the inferior boundary of the lower eyelid, which is maintained by fixed anatomical anchoring structures such as the orbicularis retaining ligament [16]. Interestingly, these lower eyelid changes were also more consistent with a broader pattern of contour remodeling rather than localized filling effects. The formation of a more globally flattened infraorbital contour suggests that highly diffusible materials may influence tissue behavior over a wider area. However, this interpretation remains exploratory and requires further validation.

This study has several limitations. First, the small sample size limits statistical power and precludes broad generalization of the findings. Second, the analysis relied on two-dimensional standardized photography, which cannot fully eliminate variability related to facial expression or lighting conditions across time points. Third, changes in infraorbital fat were inferred from surface measurements without direct radiological confirmation. The imaging findings provided in the study identify patterns that warrant further investigation in larger prospective studies incorporating imaging modalities such as dynamic ultrasound, MRI-based soft tissue motion analysis, or elastography. These modalities may allow more precise evaluation of tissue movement and stiffness changes following treatment.

In addition, the observed changes are likely multifactorial rather than attributable to a single mechanism. Early effects may be influenced by transient inflammatory responses and tissue swelling, whereas longer-term outcomes may reflect collagen remodeling processes over time [17,18]. Therefore, longer follow-up studies are needed to evaluate the durability and evolution of these effects.

Despite these limitations, this study provides a quantitative assessment of structural and functional changes following non-surgical periorbital treatment and suggests that the effects of liquid-type PCL may extend beyond simple volumetric augmentation. However, these interpretations should be considered hypothesis-generating, and further studies with larger cohorts and mechanistic analyses are required to confirm these observations.

Although the clinical effects observed in this pilot study were more limited than those typically achieved with surgical procedures such as blepharoplasty, the periorbital region remains a challenging area for non-surgical rejuvenation. Given the limited number of minimally invasive options available for this delicate region, liquid-type PCL may represent a potentially useful complementary strategy for selected patients, particularly those who are not candidates for surgery or who prefer less invasive treatment. If future studies confirm its broader regional tissue effects, this approach may help address both structural and functional aspects of periorbital aging.

## 5. Conclusions

In this pilot study, liquid-type PCL-based treatment was associated with measurable structural and functional changes in the periorbital region over three months. Improvements in upper eyelid opening were observed despite the absence of direct upper eyelid injection, and concurrent changes in lower eyelid contour parameters were also noted, suggesting indirect regional effects through contiguous anatomical planes. Furthermore, the observed concurrent reduction in UC, PP, and LC is more consistent with global remodeling of the lower eyelid contour than with localized volumization of a discrete anatomical region.

These findings suggest that liquid-type PCL treatment may function not merely as a conventional volume augmentation agent but as a functional modulator of the tissue environment governing the brow–eyelid complex and surrounding soft tissues. This raises the possibility that mechanisms beyond simple structural augmentation, such as changes in tissue interaction or distribution, may warrant consideration in the development of future periorbital rejuvenation strategies. However, validation in larger patient cohorts with extended follow-up is required.

## Data Availability

The data supporting the findings of this study are available in the Supplementary Information of this article.

## Supplementary Materials

The following supporting information can be downloaded at: https://www.mdpi.com/article/10.3390/jcm15114303/s1, Method S1: Measurement reliability analysis; Figure S1: Cannula insertion points and injection directions for periorbital treatment; Figure S2: Measurement landmarks and definitions of periorbital parameters; Figure S3: Changes in lower eyelid contour parameters in maximal state. Table S1:Changes in key periorbital parameters from baseline to 3 months; Table S2: Intra- and inter-rater reliability of periorbital measurements under neutral and maximal conditions.

## Abbreviations

PCL	Polycaprolactone
MRD1	Margin reflex distance 1
MRD2	Margin reflex distance 2
BLD	Brow-to-lid distance
CBVD-M	Medial canthus-to-brow vertical distance
CBVD-L	Lateral canthus-to-brow vertical distance
UC	Upper concavity height from lower lid margin
PP	Peak protrusion height from lower lid margin
LC	Lower concavity height from lower lid margin
CTA	Canthal tilt angle
ICC	Intraclass correlation coefficient
