# Supplementary material for: Periorbital Structural and Functional Modulation Following Liquid-Type Polycaprolactone Treatment: A Pilot Study
Source: J Clin Med. 2026 Jun 2;15(11):4303. doi: 10.3390/jcm15114303 (PMC13258640; doi:10.3390/jcm15114303)
Supplement: Supplementary file 1 [file jcm-15-04303-s001.zip › jcm-4288150-supplementary.pdf]

Supplementary Method S1

Measurement reliability analysis

To evaluate measurement reliability, intra- and inter-observer reproducibility were assessed using the intraclass correlation coefficient (ICC) based on a two-way random-effects model with absolute agreement for single measurements—ICC (2, 1). Repeated measurements were independently performed using the same image set. For inter-observer reliability assessment, measurements were additionally performed by an independent board-certified dermatologist who was blinded to the temporal sequence of the clinical photographs. Statistical analyses were performed using GraphPad Prism version 8 (GraphPad Software, San Diego, CA, USA).

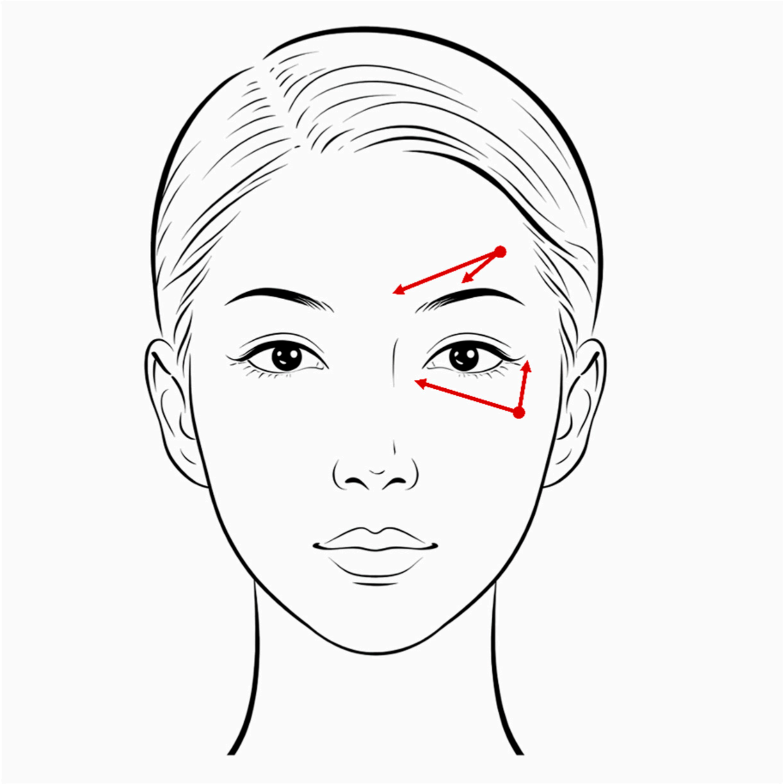

Supplementary Figure S1. Cannula insertion points and injection directions for periorbital treatment. A 23-gauge cannula was used. Entry points were established in the forehead (superior approach) and infraorbital region (inferior approach). From the superior entry, the cannula was advanced toward the brow within the subgaleal loose areolar plane. From the inferior entry, the cannula was advanced from lateral to medial and from inferior to superior along the infraorbital plane.

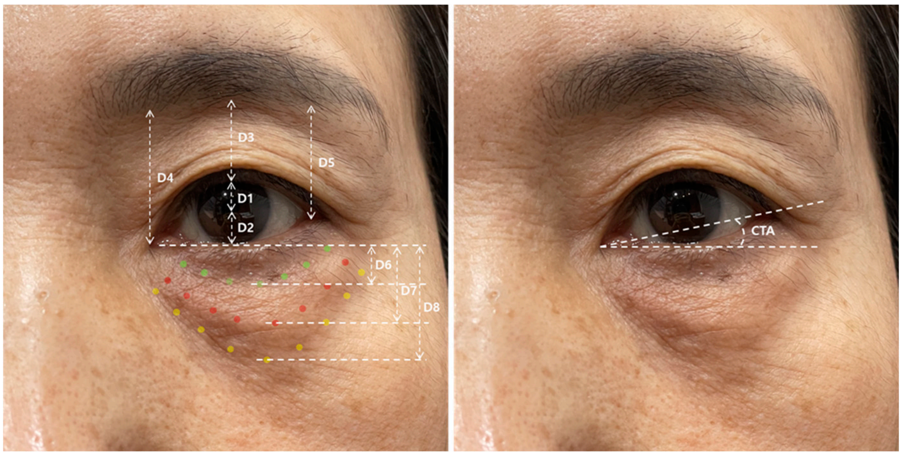

Supplementary Figure S2. Measurement landmarks and definitions of periorbital parameters

D1; Margin reflex distance 1 (MRD1)
D2; Margin reflex distance 2 (MRD2)
D3; Brow-to-lid distance (BLD)
D4; Medial canthus-to-brow vertical distance (CBVD-M)
D5; Lateral canthus-to-brow vertical distance (CBVD-L)
D6; Upper concavity height from lower lid margin (UC)
D7; Peak protrusion height from lower lid margin (PP)
D8; Lower concavity height from lower lid margin (LC)
CTA; Canthal tilt angle (CTA)

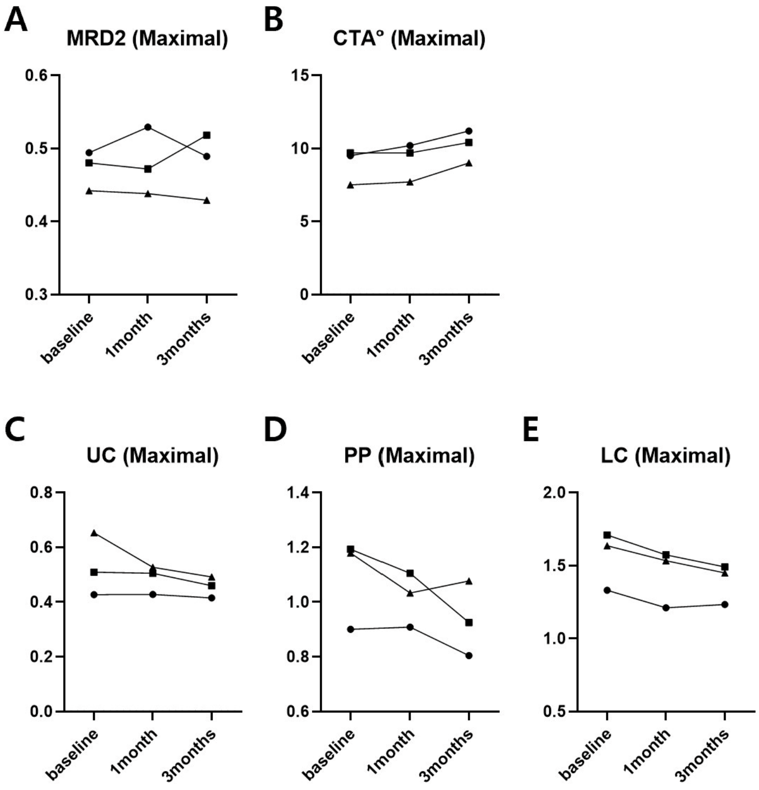

Supplementary Figure S3. Changes in lower eyelid contour parameters in maximal state. (A) Margin reflex distance 2 (MRD2), **(B)** canthal tilt angle (CTA), (C) upper concavity height (UC), **(D)** peak protrusion height (PP), and **(E)** lower concavity height (LC), measured at baseline, 1 month, and 3 months. All distance values were normalized to iris diameter. Individual cases are represented using different markers: squares for Case 1, circles for Case 2, and triangles for Case 3.

Supplementary Table S1. Changes in key periorbital parameters from baseline to 3 months. (A) Values represent absolute changes (Δ) from baseline to 3 months and corresponding percentage changes for each case. Mean values are presented with standard deviations (SD) to reflect inter-individual variability. Positive values indicate an increase, and negative values indicate a decrease compared to baseline. MRD1, marginal reflex distance 1; CTA, canthal tilt angle; UC, upper concavity; PP, peak protrusion. Measurements were obtained under standardized photographic conditions, and all values were normalized using iris diameter.

Parameter	Case 1 Δ (%)	Case 2 Δ (%)	Case 3 Δ (%)	Mean Δ ± SD (%)
MRD1 (neutral)	+0.059 (25.0%)	+0.044 (14.4%)	+0.039 (13.5%)	+0.047 ± 0.010 (17.6 ± 6.4%)
MRD1 (maximal)	+0.054 (12.6%)	+0.038 (9.2%)	+0.028 (5.2%)	+0.040 ± 0.013 (9.0 ± 3.7%)
Δ MRD1	−0.006 (−7.3%)	−0.005 (−2.6%)	−0.011 (−9.3%)	−0.007 ± 0.003 (−6.4 ± 3.4%)
CTA	+1.5 (16.6%)	+2.0 (20.4%)	+2.5 (36.2%)	+2.0 ± 0.50 (24.4 ± 10.4%)
UC	−0.190 (−36.8%)	−0.120 (−21.5%)	−0.138 (−17.5%)	−0.149 ± 0.036 (−25.3 ± 10.2%)
PP	−0.145 (−19.8%)	−0.118 (−23.0%)	−0.079 (−8.8%)	−0.114 ± 0.033 (−17.2 ± 7.4%)

Supplementary Table S2. Intra- and inter-rater reliability of periorbital measurements under neutral and maximal conditions. Reliability was assessed using a two-way random-effects model (ICC (2,1), absolute agreement). Values are presented as intraclass correlation coefficients (ICC) with 95% confidence intervals (CI) shown as [lower, upper]. All confidence intervals are rounded to three decimal places.

Variable	Intra-rater Reliability (ICC 2,1)	95% CI	Inter-rater Reliability (ICC 2,1)	95% CI
MRD1 (Neutral)	0.948	[0.772, 0.987]	0.946	[0.757, 0.986]
MRD1 (Maximal)	0.903	[0.642, 0.978]	0.897	[0.618, 0.977]
MRD2 (Neutral)	0.944	[0.763, 0.986]	0.83	[0.374, 0.955]
MRD2 (Maximal)	0.828	[0.372, 0.954]	0.792	[0.308, 0.949]
BLD (Neutral)	0.928	[0.821, 0.972]	0.7755	[0.237, 0.939]
BLD (Maximal)	0.895	[0.584, 0.974]	0.8876	[0.564, 0.972]
CBVD-M (Neutral)	0.966	[0.912, 0.987]	0.9255	[0.715, 0.983]
CBVD-M (Maximal)	0.961	[0.821, 0.990]	0.8904	[0.553, 0.971]
CBVD-L (Neutral)	0.958	[0.809, 0.989]	0.8584	[0.462, 0.963]
CBVD-L (Maximal)	0.926	[0.680, 0.981]	0.77	[0.267, 0.943]
CTA (Neutral)	0.946	[0.787, 0.988]	0.881	[0.553, 0.971]
CTA (Maximal)	0.965	[0.839, 0.991]	0.915	[0.686, 0.978]
UC (Neutral)	0.961	[0.832, 0.991]	0.955	[0.811, 0.989]
UC (Maximal)	0.962	[0.822, 0.990]	0.948	[0.788, 0.987]
PP (Neutral)	0.964	[0.838, 0.991]	0.926	[0.678, 0.981]
PP (Maximal)	0.952	[0.781, 0.988]	0.934	[0.710, 0.983]
LC (Neutral)	0.889	[0.624, 0.977]	0.864	[0.587, 0.974]
LC (Maximal)	0.909	[0.656, 0.979]	0.882	[0.575, 0.973]
